# The Relational Fit in Organizational Interventions—What Can Organizational Research Learn from Research in Psychotherapy?

**DOI:** 10.3390/ijerph18158104

**Published:** 2021-07-30

**Authors:** Malene Friis Andersen, Karina Nielsen, Jeppe Zielinski Nguyen Ajslev

**Affiliations:** 1 National Research Centre for the Working Environment, Department of Psychosocial Work Environment, 2100 Copenhagen, Denmark; jza@nrcwe.dk; 2 Institute of Work Psychology, Management School, University of Sheffield, Sheffield S10 1FL, UK; k.m.nielsen@sheffield.ac.uk

**Keywords:** organizational interventions, evaluation, effect, psychotherapy, implementation, mental health, relational fit

## Abstract

There is a growing interest in organizational interventions (OI) aiming to increase employees’ well-being. An OI involves changes in the way work is designed, organized, and managed. Studies have shown that an OI’s positive results are increased if there is a good fit between context and intervention and between participant and intervention. In this article, we propose that a third fit—the Relational Fit (R-Fit)—also plays an important role in determining an intervention’s outcome. The R-Fit consists of factors related to (1) the employees participating in the OI, (2) the intervention facilitator, and (3) the quality of the relation between participants and the intervention facilitator. The concept of the R-Fit is inspired by research in psychotherapy documenting that participant factors, therapist factors, and the quality of the relations explain 40% of the effect of an intervention. We call attention to the importance of systematically evaluating and improving the R-Fit in OIs. This is important to enhance the positive outcomes in OIs and thereby increase both the well-being and productivity of employees. We introduce concrete measures that can be used to study and evaluate the R-Fit. This article is the first to combine knowledge from research in psychotherapy with research on OIs.

## 1. Introduction

In recent years, there has been a growing interest in organizational interventions (OIs) aiming to increase the well-being of employees [1,2,3]. This interest derives, at least partly, from knowledge that poor well-being is costly for organizations and societies [4,5] and is related to poor life quality for the individual [6,7]. OIs can be defined as planned, behavioral, theory-based actions that aim to improve employee health and well-being by changing the way work is designed, organized, and managed [8]. The growth in OIs has been accompanied by an increasing interest in how to evaluate OIs [8]. Until recently, most evaluations have focused on the intervention’s effects [9]; however, there is an increasing interest in processual factors and conditions affecting the implementation and effect of OIs [2,3]. Process evaluations of OIs have revealed factors and conditions that should be taken into consideration to understand why OIs succeed or fail and why the same type of interventions can have different outcomes across organizational settings [10]. One explanation for this is that no two organizations are alike when it comes to factors and conditions such as organizational structures, tasks, change readiness, possibilities, and challenges [11]. But some under-researched factors that are of importance to the outcome of an OI exist. In this paper, we argue that the person or persons that drive and facilitate the OI—labeled Intervention Facilitator (IF) in this article—also affect the outcome of an OI, that this also goes for the participants in the intervention, and not least for the relation between the IF and the participants in the intervention. In this paper, we present a theoretical framework for how researchers may understand the role of IFs and the relation between the IFs and the participants in an OI. This is a conceptual paper that integrates research and theory from the fields of both OI and psychotherapy, as the research field in psychotherapy can teach us much about which components in an intervention especially affect its outcome. We hope that our conceptual paper will inspire future empirical evaluations of OIs. In order to enhance the chances for this, we introduce modified concrete measures that can be used to study, validate, or reject the theses presented here.

### The Importance of Fit in Organizational Interventions

Frameworks for the process evaluation of OIs identify three central themes in an appropriate process evaluation: (1) the intervention design and implementation, (2) the intervention context, and (3) participants’ mental models of the intervention and their work situation [12]. Recently, it has been suggested that OIs are more likely to bring about intended outcomes if there is a good “fit” between context and intervention as well as a good fit between the intervention and the persons participating in the intervention [13]. The concept of person–intervention fit stems from the organizational psychology theory of Person–Environment fit (P–E fit), which has been defined as the “compatibility between an individual and a work environment that occurs when their characteristics are well-matched” [14].

In relation to organizational interventions, fit includes the need to match and adjust the intervention to existing practices and procedures within organizations and the need to consider, for example, participants’ perceived readiness for change and educational levels. One type of fit is the Person–Job fit (P–J fit), which concerns the fit between individuals’ skills and competencies and the demands of the job [15]. In a review of OI processes, Nielsen and Noblet called for studies on the role of the IF as well as on the relation between the IF and the participants [16]. This call is echoed in recent debates focused on the role of facilitation and facilitators within implementation science [17,18].

Although the importance of the IF has been considered, the role of IFs and the degree to which they influence the implementation and effect of an intervention is not well described in existing literature on OIs [16]. To the best of our knowledge, only one study has focused on the fit between facilitators in organizational interventions and their skills and knowledge. In a study where employees were appointed facilitators within their own departments, the researchers found that those facilitators who were dissatisfied with their jobs but felt they possessed the necessary competencies to fulfil the facilitator role reported higher levels of job satisfaction post-intervention [19]. In the present paper, we answer these calls by proposing a framework for examining how IFs, the participants in the intervention, and the relation between the IF and the participants may affect the outcome of an OI. We suggest that future evaluation research on OIs should look systematically into what we conceptualize as the Relational Fit, i.e., the fit between the IFs and the participants. This knowledge is important to be able to develop a more thorough understanding of why OIs fail or succeed in bringing about the intended outcomes: improving the work conditions and health of employees.

Our conceptualization of the relational fit is based on research studies on OIs and the well-established research field of psychotherapeutic interventions. For decades, the psychotherapeutic field has systematically explored the importance and effect of relations in therapeutic interventions, and we believe it may confer knowledge that could help researchers in OIs to evaluate relational fit in future studies. By integrating knowledge from research on psychotherapy with research on OIs, we present important factors in relational fit in OIs. Inspired by measures used in psychotherapy, we provide concrete suggestions for how future studies may explore and evaluate the relational fit and its impact on the outcome of OIs. Our inspiration to use the concept of “relational fit” is derived from research on the two above-mentioned fits. But the term “fit” is also used—although rarely—in research in psychotherapy regarding the fit between patient and treatment. In this article, we therefore incorporate the knowledge from psychotherapy into the concept of “fit” derived from the research on OIs.

**Figure 1:**
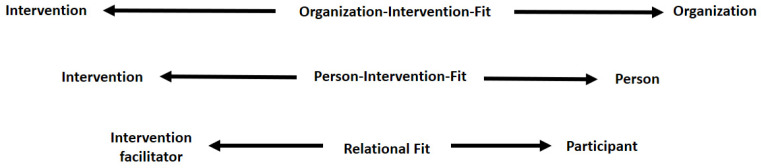
Three important fits in an Organizational Intervention (OI).

The relational fit should be considered as an extension of the two fits presented by Nielsen and Randall [13]: context-intervention and person-intervention, as shown in Figure 1.

As can be seen in Figure 1, we use the term “participant” instead of “person”, which is the term used by Nielsen and Randall [13]. The reason for this is that we find the term “person” too broad for the purpose of our article, and we want to emphasize that we focus on and write about two different players: those who participate in the intervention and those who facilitate it.

This paper is a first step in bringing together the research fields of OI and psychotherapy. We first introduce major findings from research in psychotherapy, which highlight the importance of common factors for positive outcomes in therapeutic interventions. Second, we unfold the relational fit between IFs and participants in OIs by presenting key findings from research in psychotherapy and OIs. Instead of presenting the major findings from each field in separate sections, we have chosen to integrate the major findings from both fields in the following three sections: (1) Intervention facilitators, (2) Participant factors, and (3) Quality of relation. In this way, we hope to give the best introduction to what might be the most effective components in both psychotherapy and OIs.

Our ambition is that future empirical studies will test our framework and explore the effect and importance of the relational fit in OIs and thereby increase the chances for positive outcomes in OIs

## 2. What Affects the Outcome of Psychotherapy?

Back in 1961, Jerome Frank published his famous book Persuasion and Healing in which he argued that change in patients during psychotherapy occurs when factors that are common to all forms of psychotherapy operate in concert [20]. Frank’s work has been a great inspiration for research in understanding what affects the outcome of psychotherapy. A number of psychotherapeutic traditions exist, each of which has their own therapeutic methods and techniques (e.g., psychodynamic therapy, cognitive behavioral therapy, etc.) [21]. Despite variations, these methods and techniques share some common characteristics: there is a therapist, a client (or a group), and a relation between the two. These common characteristics are labelled common factors and refer to aspects of psychotherapy that are important in all therapeutic schools: a good therapist, an engaged client, and a high-quality relation between therapist and client [22].

Through decades of studies, researchers in psychotherapy have refined their evaluation methods to achieve more detailed knowledge of the extent to which different intervention components can explain the effects of a therapeutic invention [23,24]. Research has revealed that specific therapeutic methods and techniques can explain only approximately 15% of the effect of the intervention (e.g., reduction in symptoms, increase in quality of life), while extra-therapeutic changes (spontaneous remission, events that are not related to the therapy) account for 40% of the effect. The clients’ motivation and expectations of the therapy can explain 15% of the success or failure of the therapeutic intervention. The remaining 30% can be ascribed to common factors (therapeutic alliance and therapist characteristics) [23,24,25]. In other words, common factors account for a greater portion of the effect than the specific therapeutic method and is one of the strongest predictors of success or failure in psychotherapy [22,26].

Although there are differences between OIs and psychotherapeutic interventions, they also share a number of features: they both aim to change the thoughts and actions of individuals and/or groups, they both enhance well-being, and they both address patterns and dynamics at the individual and group levels in order to create awareness of behavior that facilitates or hinders well-being [27,28,29,30].

Even though the relation between IFs and participants in OIs might not be as deep or intimate as the relation between therapists and clients, we argue that the relation also plays a central—but so far widely neglected—role in the implementation and outcome of an intervention.

## 3. The Relational Fit in OI

Translating the insights from the research on common factors in psychotherapy, it is important to discuss three different but interrelated components of the relational fit. First, as in the case with the therapist, the IFs’ competences and abilities may influence how the OI is implemented and in what way participants are involved in the intervention. Second, similar to the client, participants’ expectations, self-efficacy, experiences, etc., may affect their motivation and ability to participate in and use the intervention, as well as their motivation and ability to integrate the intervention in their work. The third is the quality of the relation between IFs and participants, as the quality of this relation can influence the motivation of participants for engaging actively in OIs. In the following presentation of the literature, we have chosen to mainly include reviews or meta-analyses that give an overview of the literature on common factors. Thus, we have not included primary studies in the following unless there are no existing reviews or meta-analyses on the relevant subjects.

### 3.1. Intervention Facilitators

Even though there is almost always an IF who facilitates and implements the intervention in cooperation with the participants, very little is known about how the IFs influence the success or failure of such interventions. However, a few studies have pointed out relevant competencies and roles for the IF. Nielsen et al. suggested that IFs should “possess expertise in process consultation and knowledge about occupational health issues” [31], and Peiró et. al. (2007) in Nielsen et al. pointed to the following competencies that external consultants should embrace: change management skills, expert knowledge of psychosocial risk factors, awareness of regulations and laws, and practical skills in conducting risk assessment and evaluation [31]. A few studies in OIs have suggested that IFs’ communication skills are important as these could influence participants’ perception of the motives and objectives of the intervention—and thereby their commitment to engage in intervention activities [12,32,33,34]. To the best of our knowledge, no studies have looked systematically and empirically into the above-mentioned aspects of evaluation for OIs, even though especially qualitative process evaluations of OIs have shown that participants perceive IFs as important for the implementation of the OI [3].

Another unanswered question in the literature is whether internal or external IFs may be more suited for supporting OIs. Former research has shown that the IF role has usually been managed by external or internal consultants, or the manager [12,33,35], but we know little about the pros and cons of the IF being a manager, an external consultant, or an internal consultant [35,36]. It has been suggested that external consultants may be more objective and avoid taking sides [37], but others have argued that internal IFs might more successfully sustain the possible positive effect of the OI [33,35]. Berta et al. [17], Semmer [10], and Nielsen and Randall [13] called for systematic research into the role of IFs in OIs, and Nielsen has called for more research into how managers as IFs can “make or break an intervention” [33].

In pursuing a more systematic evaluation of the role of the IF, inspiration may be drawn from research in psychotherapy as this research field has already looked into this. In their book “The Great Psychotherapy Debate”, Wampold and Imel write that we need to look at therapists in the same way as we do other professionals: some lawyers win more cases than others and some teachers inspire their students to achieve better grades than others. Therefore, researchers should continually explore what characterizes the more successful therapist so others can learn from them [38]. We argue that the same goes for IFs: if we want to improve the effect of OIs, we need to know what characterizes the most successful IFs.

It is well-documented that the therapist’s empathy enhances the chances of clients profiting from individual psychotherapy [30,39]; Kivligham et al. have also found that therapists’ level of empathy increases the group members’ level of engagement in group therapy [40]. Furthermore, in a review, Ackerman and Hilsenroth identified 11 therapist characteristics and attributes as well as 11 therapist generic techniques (how the therapist more specifically helps the client to reflect and change thoughts and behavior) that affect the therapeutic alliance and outcome of an intervention [41]. The 11 personal characteristics and attributes are: flexible, experienced, honest, respectful, trustworthy, confident, interested, alert, friendly, warm, and open; the 11 therapist techniques related to the characteristics and attributes are: exploration, depth, reflection, supportive, notes past therapy success, accurate interpretation, facilitates expression of affect, active, affirming, understanding, and attends to clients’ experience. Whereas the existing OI research has suggested that IFs’ knowledge- and communication-related competencies are important, psychotherapeutic evidence has also documented that relational and sociable characteristics are important in increasing the effect of an intervention. To provide a starting point for research on the importance of these characteristics to the outcome of OIs, we adapted Ackerman and Hilsenroth’s therapist characteristics and techniques [41] in the formulation of 11 questions for the mapping of the IF’s characteristics and techniques (Table 1). We have prepared 11 questions aimed at identifying whether participants in OIs feel and experience that IFs possess and apply these techniques in their implementation and facilitation practice.

The qualities outlined above may not necessarily be equally important for Ifs. However, we suggest that asking these questions may provide a starting point for future quantitative as well as qualitative explorative research on the role of IFs in the OI.

### 3.2. Participant Factors

It has been suggested that the following three participant factors may play a role in the implementation and effect of OI: (1) mental models, (2) self-efficacy, and (3) readiness for change. Research on OIs points to the importance of participants’ mental models of (1) the intervention program and (2) the intervention activities and how they influence the motivation to participate in the intervention [2,42]. Participants’ mental models are important as they may influence how participants understand and react to the intervention and how they perceive their own and the management’s responsibility in the working environment [12,43]. It has also been shown in qualitative process evaluations how participants’ views on the feasibility and acceptability of the intervention method influence their motivation to implement the intervention [2,3]. Participants’ self-efficacy (how much one believes in one’s competencies) is hypothesized to affect the outcome of an intervention, as the level of self-efficacy can influence the employees’ belief in their own capability to engage actively in the intervention and implement the suggested improvement of working conditions [44]. Furthermore, it is suggested that participants’ readiness for change may influence how motivated they are for participating in the intervention [12,31]. As can be seen, what in therapy is labeled “participant factors” has, to a certain degree, been the topic of investigation in OI research. But there is no tradition for systematically addressing this in the evaluation and understanding of the outcome of OIs.

While researchers in OIs have only suggested the importance of participant factors [13], researchers in psychotherapy have both theoretically and empirically studied which individual factors and variables affect outcomes [22,26]. We suggest that several aspects drawn from psychotherapeutic research may hold explanatory value and could be incorporated in future evaluations of OIs. First, research in psychotherapy has documented a relation between the client’s expectations of therapy and the outcome [30,45,46]. Second, client factors such as openness [47], motivation, and the ability to establish stable relationships, to verbalize, and to cooperate lead to more successful outcomes [48]. Third, the client’s attachment style and social competencies influence the client’s ability to develop a strong alliance and therefore indirectly influence how much the client profits from the intervention [49].

More than 161 patient characteristics have been studied to investigate the extent they can affect psychotherapy outcomes [39]. As one of the most well-documented patient characteristics is the patients’ expectations and their belief that the therapy will work [30,39], we utilized the Credibility/Expectance Questionnaire (CEQ) developed by Devilly and Borkovec [50] as a main source of inspiration for measuring the effect participants have on the outcome. The CEQ is widely used in contemporary psychotherapy research and has been shown to account for approximately one-third of the variance in treatment outcomes [45,51]. In order to make the CEQ operational for OIs, we present our reconceptualization in Table 2. We have modified the original questions from the CEQ and adjusted them for OIs. As can be seen in Table 2, we have chosen to replace “feel” with “think”, as this term may have a higher face validity and may be more recognizable for participants in OIs. We hope that future studies of OIs will use, validate, and qualify our suggestions.

Applying this modified instrument along with a qualitative exploration of participants’ expectations of and motivation for OIs may provide useful insights into which different types of OIs and IFs fit different participant groups best. Knowledge derived from this type of analysis will enable the matching of both the intervention and the IFs with the participants’ needs and conditions.

### 3.3. Quality of Relation

Studies on OIs have highlighted that interventions may fail to achieve their objectives if participants and IFs disagree on which work-related problems the intervention should address, what the goal of the intervention is, and/or which methods should be used in the intervention [52,53]. Furthermore, an OI may fail to achieve its intended outcome if there is considerable distance between participants’ and IFs’ perceptions of how work-related problems should be addressed [54]. However, none of these factors are included in current evaluation frameworks [12,43].

Research in psychotherapy has systematically documented that the relation influences the outcome of the therapy, and that the quality of the relation shapes the client’s positive or negative mental models of the psychotherapeutic intervention [22,24,55]. A strong alliance in psychotherapy is characterized by a positive and respectful emotional bond, by appreciation, mutual agreement on treatment goals, and a feeling that problems are addressed and managed in relevant ways [30,46,56,57]. From research in psychotherapy, we know that a strong alliance and particularly agreement on the tasks and goal of the therapy increase the chances of the client engaging in healthier actions and improving their well-being [30]. A strong alliance increases the likelihood of the client accepting the intervention and being confident that treatment has a positive effect [30,58]. Research has shown that the therapist’s contribution to the alliance has a greater impact on the outcome than the client’s contribution [21], and a meta-analytic review concluded that the quality of the alliance is associated with the client’s perceptions of the therapist’s empathy and genuineness [57].

We propose that an evaluation of the emotional bond, appreciation, and mutual agreement on methods and goals may likewise be important for researchers in OIs. In this regard, the Working Alliance Inventory (WAI) presents a well-established paradigm and may also be fruitful in the evaluation of OIs. WAI consists of 36 survey items responded to by both participants and therapists and has shown consistent predictive capacity in relation to counseling outcomes [26,59]. In recent years, the WAI has been successfully modified to fit other areas of therapy or counselling such as physiotherapy, stuttering treatment, and physical rehabilitation [60,61,62]. The WAI may also—in a modified version—be transferable to investigating the quality of the relation between the IF and participants in OIs. Since the WAI in the original form consists of 36 items, it may be too long for participants in OIs to complete. Hence, in Table 3 we suggest a modification, taking its departure from Hatcher and Gillaspy’s [63] revised short version of the WAI. This version has validated the use of 12 items of the WAI, with the highest capability to predict client outcomes. We have modified the original questions from the Working Alliance Inventory—Short Revised (WAI-SR) Subscales and adjusted them to a work context and to an organizational intervention.

We suggest that these items may provide a starting point for studying the importance of the quality of the relation between the IF and the participants in OIs.

## 4. Discussion

In the present paper, we introduced a new important fit—the relational fit—in OIs. The relational fit focuses on the characteristics, attributes, and techniques of IFs and participants as well as on the quality of their relation. The relational fit complements existing “fits” literature (organization–intervention fit and person–intervention fit) in OI research [13]. We showed how research on OIs can benefit from psychotherapeutic research in understanding and exploring the importance of IFs and the relational fit between IFs and participants; we also proposed that this can help us understand why interventions succeed or fail. Furthermore, we suggested measures that may be used to systematically evaluate relational fit in future studies. Such evaluations may provide valuable insights into designing the OIs and also help us to prepare the IFs for the running and implementing of OIs. We proposed that evaluations of the relational fit between IFs and participants in OIs may take inspiration from three adjusted surveys and measurement methods presented in this paper (1) Ackerman and Hilsenroth’s review of therapist characteristics and techniques, (2) Devilly and Borkovec credibility/expectancy questionnaire (CEQ), and (3) The Working Alliance Inventory—Short Revised (WAI-SR) Subscales), which were developed for use in psychotherapy. We also argued that adjusted versions of these could be a good starting point. In this article, we focused on adjusted quantitative questionnaires. It might, however, also be relevant to conduct qualitative research on the presented subjects. The advantage of the quantitative methods here is that they allow the relational fit to be analyzed with outcome variables [64].

A study has shown that IFs who had undergone training in how to manage and implement an intervention increased the positive effect of an OI more successfully than IFs who did not receive training [65]. Knowledge on the relational fit and important IF competences and techniques could be useful in the optimal training of Ifs, with special focus on their ability to develop good relations with the participants, thereby potentially increasing positive outcomes of OIs. Knowledge on which participant factors and to what degree these affect the relation to the IF will also help to better match IFs and participants, which could also help the IF to meet the needs and conditions of the participants.

Our arguments for the importance of a high-quality relation between the IF and participants in OIs go hand in hand with one of the main findings and general recommendation in the literature on intervention method in OIs, namely that OIs should be participatory [66,67]. This recommendation has frequently been voiced, as participatory interventions seem to be most successful in improving the working environment and the health of employees [31]. The participatory element in these approaches is highlighted as securing the relevance of interventions through the activation and consideration of the practical knowledge and know-how of managers, employees, and other potentially relevant actors [68]. Qualitative studies have also pointed to the importance of the IF in participatory interventions. Conversation analyses of the dialogue between IF and participants showed that the IF’s way of facilitating the intervention had an effect on whether the participants participated actively or were passive in the intervention workshops, depending on the IFs’ facilitation styles [69,70]. Studies have shown how IFs varied in how much they welcomed the participants’ work improvement suggestions, how open they were to suggestions, and whether they themselves played an active role in supporting or rejecting the concrete suggestions [69,70]. We therefore propose that a high-quality relation between IFs and participants produces an increase in motivation and the willingness to actively participate in the intervention by, for example, expressing criticism of the organization and the working environment, or by presenting improvement ideas, taking risks, and suggesting changes. A high-quality relation could therefore be important and even necessary to unleash the potential of participation as a working mechanism in OIs.

In this paper, we argued that the research field of psychotherapy may bring us closer to answering the call from Nielsen and Randall for the development of evaluation models and methods that can “identify how the potential effects of interventions on health and well-being are moderated and mediated by intervention processes” (p. 602) [12]. The degree to which the results and measurement methods from psychotherapy are transferable to organization interventions, and whether common factors have the same explanatory power in OIs have yet to be explored. Therefore, we hope that researchers will be inspired to look more systematically into the relational fit in future evaluations of OIs. Knowledge on relevant and important characteristics, attributes, and techniques of IFs would be of great relevance to practice, as it may permit the training of IFs and facilitate their ability to develop relations of high quality, thereby increasing the chances of the OIs’ success. We argue that knowledge on the importance of a good relational fit will help decision makers when planning and deciding on an OI to improve employees’ well-being. They need to be aware that it is insufficient to select an evidence-based intervention that fits the organization. It may be just as important—or even more important—to select highly qualified and trained IFs as well as to continuously ensure that there is a good fit between the IF and the employees participating in the intervention.

## 5. Conclusions

Many organizations initiate OIs to improve employees’ mental well-being. From research, we know that the effect of these interventions is limited and not sufficient for researchers and organizations to be able to choose and implement an evidence-based intervention. In this article, we have argued that it might be equally important to secure a good fit between the person or persons facilitating the intervention and the employees participating in the intervention. We labelled this fit the Relational Fit. Based on research from the fields of OI and psychotherapy, we have shown the importance of future studies addressing and evaluating the R-fit, and we hope that researchers will be inspired by the three concrete measures presented here to further study and evaluate the R-Fit. Knowing that there is an increase in mental health problems, it is especially important to optimize the OIs that aim at improving the mental well-being of employees. We hope that with this article, we have managed to play a small but important role in this.

## Figures and Tables

**Table 1:** (Re)conceptualization of therapist characteristics and techniques into the relational fit: IF characteristics.

Ackerman and Hilsenroth’s Review of Therapist Characteristics and Techniques		Relational Fit Items—Intervention Facilitator Characteristics and Techniques
*Personal attributes*	*Technique*	The participants are asked the following questions (we suggest using a 5-point Likert scale): To which degree do you experience that…
Flexible	Exploration	the IF contributes to investigating several sides of work-related issues
Experienced	Depth	the IF asks relevant questions to get to the center of issues
Honest	Reflection	the IF shows an interest in improving our working environment
Respectful	Supportive	the IF encourages reflections and suggestions in a respectful way
Trustworthy	Notes past therapy success	the IF is attentive to the progress made in improving the work environment
Confident	Accurate interpretation	the IF understands work-related problems and suggestions for possible solutions
Interested	Facilitates expression	the IF actively includes participants’ different perspectives
Alert	Active	the IF is actively present in the process
Friendly	Affirming	the IF is friendly and appreciative
Warm	Understanding	the IF understand us
Open	Attends to client’s experience	the IF is curious about our experiences in work-related problems

IF = Intervention Facilitator.

**Table 2:** (Re)conceptualization of the CEQ into relational fit: Participant factors.

Devilly and Borkovec: Credibility/Expectancy Questionnaire (CEQ)	Relational Fit Items—Participant Factor
How logical does the therapy offered to you seem	Are the OI’s aims and objectives clear to you?
How successfully do you think this treatment will be in reducing your symptoms	How successful do you think the OI will be in improving your working environment?
How confident would you be in recommending this treatment to a friend	How confident would you be in recommending this OI to another team or organization?
How much improvement in your symptoms do you think will occur	How much improvement in the working environment do you think will occur?
How much do you really *feel* that therapy will help you to reduce your symptoms	How much do you really think that the OI will improve your working environment?
How much improvement in your symptoms do you really *feel* will occur	How much improvement in your working environment do you really think will occur?

OI = Organizational Intervention.

**Table 3:** (Re)conceptualization of the WAI-SR into relational fit: Quality of relation.

*Working Alliance Inventory—Short Revised (WAI-SR) Subscales*	*Relational Fit Items—Quality of Relation*
*Goal Scale*	*Goal scale*
The therapist and I are working towards mutually agreed upon goals	IF supports us in working towards agreed upon goals to improve our working environment
We agree on what is important for me to work on	The IF and the team agree on what is important for us to work on
The therapist and I collaborate on setting goals for my therapy	The IF and the team collaborate on setting goals for the OI
We have established a good understanding of the kind of changes that would be good for me	The IF and the team have established a good understanding of the kind of changes in our working environment that would be good for us
*Task Scale*	*Task scale*
What I am doing in therapy gives me new ways of looking at my problem	What the IF and the team are doing in the process is giving us new ways of looking at our work-related problems and challenges in our working environment
I feel that the things I do in therapy will help me to accomplish the changes that I want	The things the IF and the team do in relation to the OI will help us to accomplish the work-related changes that we want
As a result of these sessions I am clearer as to how I might be able to change	As a result of the activities we engage in with the IF, I am clearer as to how I might contribute to the desired change in the working environment
I believe the way we are working with my problem is correct	I believe the way we are working with the IF on our work-related problems is correct
*Bond Scale*	*Bond scale*
I believe the therapist likes me	I believe that the IF likes the participants in the intervention
The therapist and I respect each other	The IF and the participants respect each other
I feel that the therapist appreciates me	I feel that the IF appreciates us
I feel the therapist cares about me even when I do things that he/she does not approve of	I feel the IF cares about us even when we do things that he/she does not approve of

IF = Intervention Facilitator, OI = Organizational Intervention.
